# Pseudo-arthrosis of the spine of the scapula: a case report with a delayed diagnosis

**DOI:** 10.1007/s11751-014-0210-2

**Published:** 2014-12-25

**Authors:** Cem Copuroglu, Levent Tan, Elif Copuroglu, Mert Ciftdemir, Mert Ozcan

**Affiliations:** 1 Department of Orthopaedic Surgery, Faculty of Medicine, Trakya University, Edirne, Turkey; 2 Department of Orthopaedic Surgery, Corlu Private SIFA Hospital, Tekirdag, Turkey; 3 Department of Anesthesiology, Faculty of Medicine, Trakya University, Edirne, Turkey; 4 Trakya Universitesi Tip Fakultesi, Ortopedi ve Travmatoloji Ana Bilim Dalı, Edirne, Turkey

**Keywords:** Pseudo-arthrosis, Scapula, Late diagnosis

## Abstract

Scapular spine fractures are rare injuries. The aim of this study was to evaluate a late-diagnosed scapular spine pseudo-arthrotic patient. Because of the surrounding soft tissue mass and overlapping of the scapula with the thoracal bones on a roentgenogram, diagnosis may be missed or delayed for years. We present a case of scapular spine pseudo-arthrosis in a 50-year-old man, who sustained a traffic accident 2 years ago. He was treated as a soft tissue injury of the left shoulder and later as a rotator cuff tear. His scapular spine fracture was diagnosed as pseudo-arthrosis of the scapular spine with a diagnostic delay of 2 years. Isolated scapular spine fractures are rare, usually associated with other injuries and frequently treated non-operatively. Sagging of the acromion as a result of a scapular spine fracture may mimic supraspinatus outlet impingement. If a painful pseudo-arthrosis limits the function of a shoulder, fractured ends should be fixed until union occurs. Although scapular spine fractures are rarely seen, they must take place in the differential diagnosis of impingement syndromes of the shoulder.

## Background

Scapular fractures are rare injuries which consist of 1 % of all fractures and 5 % of shoulder girdle fractures [1, 2]. Most of the scapular fractures are neck and body fractures, and only 6 % of scapular fractures are scapular spine fractures [3].

Injuries to the thoracic cage and soft tissues around the shoulder girdle are common and may lead to a delayed diagnosis of the scapular fracture. In this case report, we present a late-diagnosed scapular spine fracture, which has been treated conservatively for soft tissue injury and later operated for rotator cuff tear without the diagnosis of pseudo-arthrosis of the spine of the scapula.

## Case report

**Fig. 1:**
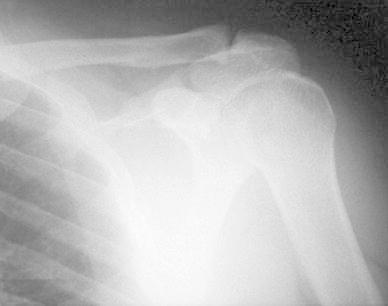
Preoperative roentgenogram, first intervention

**Fig. 2:**
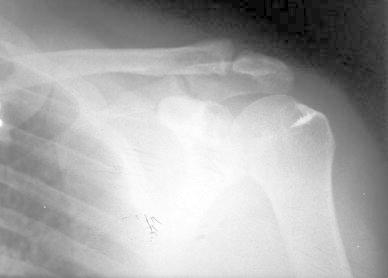
After rotator cuff repair roentgenogram

**Fig. 3:**
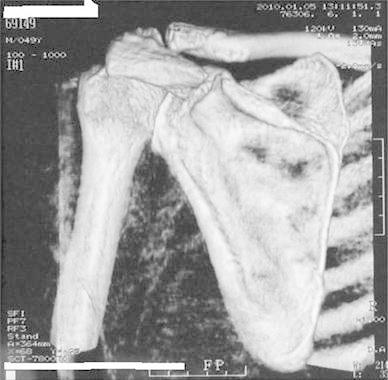
Frontal computed tomography (three dimensional)

**Fig. 4:**
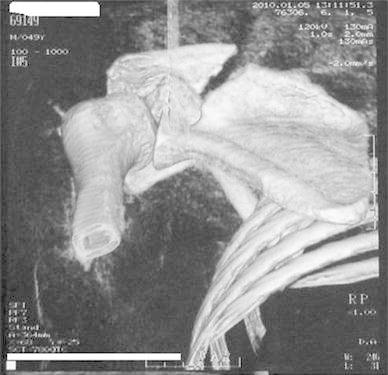
Axial computed tomography (three dimensional)

A 50-year-old right-hand dominant man with a painful left shoulder admitted to our orthopedics’ outpatient department. In his history, he had a traffic accident, a truck hit to his left shoulder while he was walking, 2 years ago. In his first intervention, he has had swelling and a large ecchymosis with tenderness at the posterior of his left shoulder and has been treated for soft tissue injury with conservative treatment modalities, at another institution (Fig. 1). His pain decreased but non-specific pain insisted on. Almost 1 year after the trauma, he had a painful sub-acromial impingement at the left shoulder. Clinical examination showed that impingement tests were positive. Magnetic resonance imaging (MRI) studies showed a complete supraspinatus tendon rupture. Because of the rotator cuff tear, he was operated and supraspinatus tendon repair and sub-acromial decompression was applied, arthroscopically. After the operation, he was followed up for physical therapy and he could not regain enough range of motion and strength. Since that trauma, he had continually suffered from moderate pain of his left shoulder. As the shoulder pain went on, he was re-evaluated in our clinic. On the physical examination, he had tenderness on the spine of the scapula with palpation. Active and passive abduction and forward flexion were painful at 90°, and internal and external rotation was comfortable. We took new radiographs of his left shoulder, and on these radiographs, we noticed the pseudo-arthrosis of the spine of the scapula at the base of the acromion and entering the spino-glenoid notch (Fig. 2). Computed tomography (CT) studies also revealed the pseudo-arthrosis of the spine of the scapula (Figs. 3, 4). After informative discussion about the operation, the patient gave informed consent for the procedure.

**Fig. 5:**
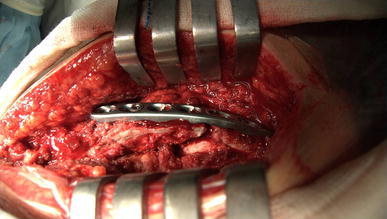
Preoperative or intraoperative

**Fig. 6:**
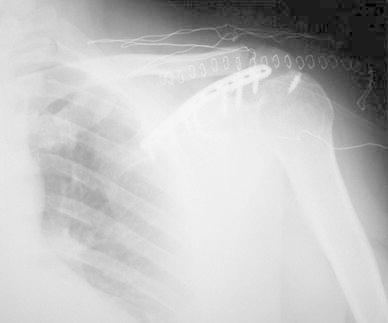
Early postoperative roentgenography

**Fig. 7:**
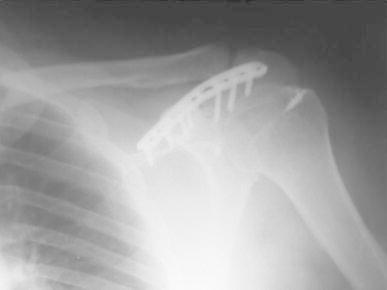
Follow-up roentgenogram, postoperative 6 months

**Fig. 8:**
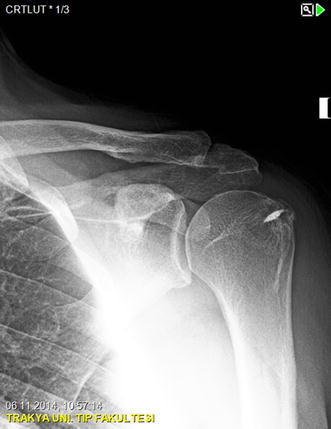
Follow-up roentgenogram, postoperative 2 years

**Fig. 9:**
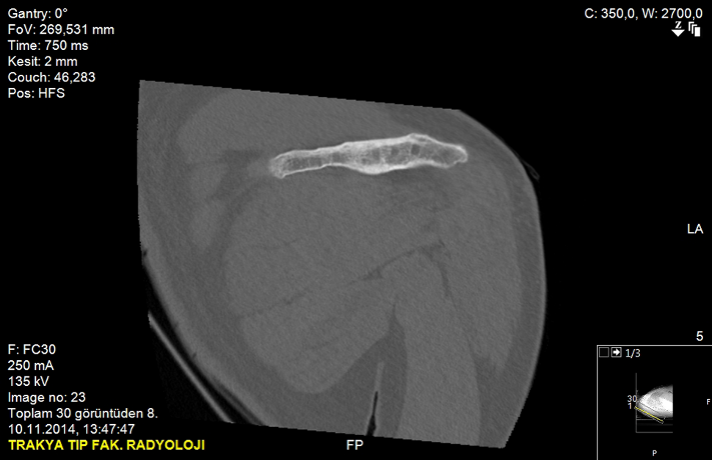
Follow-up computed tomography coronal section

**Fig. 10:**
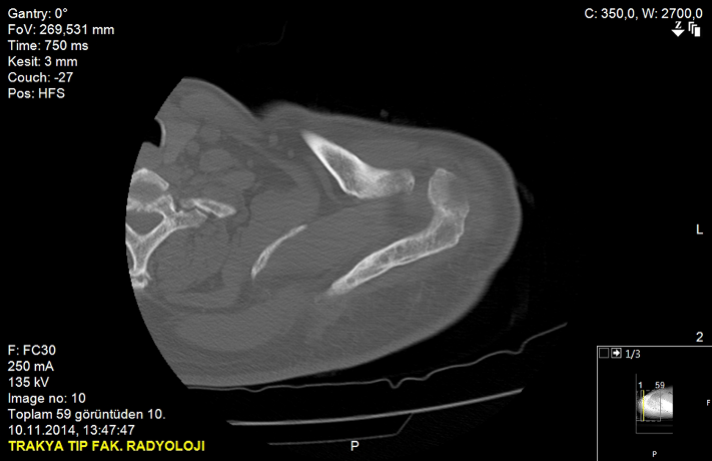
Follow-up computed tomography axial section

**Fig. 11:**
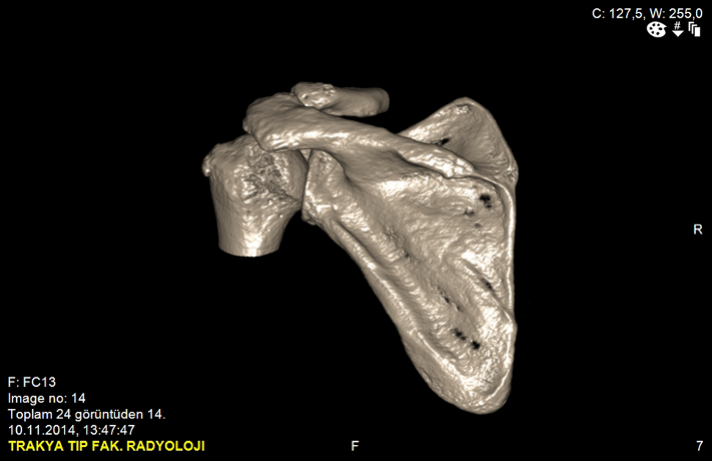
Follow-up computed tomography (three dimensional)

**Fig. 12:**
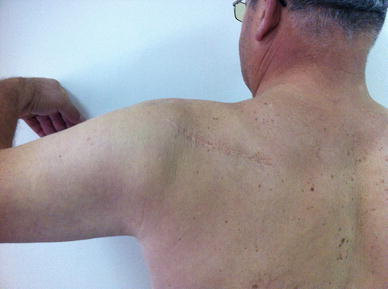
Clinical picture of the patient, postoperative 2 years

In the lateral decubitus position, the bony prominences were padded adequately; left arm and left iliac crest were prepared and draped in a usual manner. Using an incision of about 10 cm, parallel to the spine of the scapula, deltoid-trapezius fascia was opened along the line of the scapular spine using sharp dissection (Fig. 5). All atrophic tissue was removed with a curette until fresh bleeding bone was reached. Cancellous bone graft was harvested from the left iliac crest with the help of a curette. Fractured bone ends were reduced, and locking compression plate (LCP) with eight holes was implanted with autografting. High compression could be achieved because the plate was fixed very well between the two lamina of the spine of the scapula. Intraoperatively, fractured bone ends were checked with image intensifier and satisfactory medial and lateral plate fixation was obtained. The detached muscular parts were re-attached to both sides of the muscle with the help of absorbable sutures and the fascia, and the skin was sutured. After the operation, the shoulder was immobilized in a Velpeau bandage for a week. Passive exercises were begun in the beginning of the second week, and active-assisted exercises were started in the beginning of the fourth week, postoperatively. Three weeks later, at the beginning of the seventh week, active range-of-motion exercises were started. Twelve weeks postoperatively, roentgenograms showed limited bony healing of the pseudo-arthrosis with satisfactory implant position (Fig. 6). Painful impingement syndrome disappeared immediately after the operation. This active man was free of pain and re-gained full motion and muscle strength. At the follow-up examination, 6 months postoperatively, the patient was still pain-free and managed to do his daily activities comfortably and the fracture had limited healing (Fig. 7). In the latest radiographs (postoperative 2 years), he had complete union of the previous non-union of the scapular spine (Fig. 8) and computerized tomography views show the complete bony union (Figs. 9, 10, 11). His visual analogue scale (VAS) score was 8 preoperatively and 2 at postoperative sixth month and 1 at postoperative 2 years. Clinical picture of the eventual result can be seen in Fig. 12.

## Discussion

Isolated fractures of the scapular spine are relatively uncommon injuries [1, 3, 4]. They are usually a result of high-energy trauma; most are non-displaced or minimally displaced and treated non-operatively [1, 2, 5]. Most scapular fractures heal with a good functional result after conservative treatment [6]. In our patient, the injury has happened as a result of direct trauma from posterior of his left shoulder by a truck bump, while he was walking. This is a high-injury blunt trauma to the dorsal aspect of the chest wall associated with obvious bruising. Scapular body is surrounded by a large soft tissue mass and needs a high-energy mechanism to cause a scapular body fracture. He has been treated non-operatively for soft tissue injury, and the diagnosis of the scapular fracture has been missed on the presentation radiographs. If such a fracture has been diagnosed in the acute setting, acute management would be simpler, so that acute management results in more healed fractures of the scapula than delayed management. Scapular spine fractures are frequently mis-diagnosed [2], leading to a diagnostic delay of many years, as reported by Böhm, 30 years of delayed diagnosis [1].

Scapular body fractures may cause weak rotator cuff function and loss of active arm elevation, named ‘pseudo-rupture of the rotator cuff,’ probably due to inhibition of muscle contractions from intramuscular hemorrhage [2]. Also, pseudo-arthrosis of the spine of the scapula or acromion, like os acromiale, predisposes to sub-acromial impingement [1]. The pull of the deltoid muscle can tilt the fragment inferiorly, which compromises the function of the rotator cuff [1, 7]. Sagging of the lateral spine and acromion effectively produces narrowing of the supraspinatus outlet and secondary impingement of the rotator cuff [3, 8]. As Lambert et al. [9] mentioned in their study, scapular spine fracture represents a partial failure of the lateral scapular suspension system, leading to a failure of scapular postural control, with resulting sub-acromial impingement.

One year before his admission to our clinic, persistent pain and significant limitation of function have made his doctors take an MRI and complete supraspinatus tendon rupture has been diagnosed on the MRI. Physical examination has been concordant with the MRI, so doctors have focused on the supraspinatus rupture. He has been operated for rotator cuff tear arthroscopically, and supraspinatus tendon repair and sub-acromial decompression have been applied.

As time passed, his complaints about his shoulder did not regress. He had limited function and pain at the posterior side of his shoulder. Two years after the trauma, when his shoulder radiographs were repeated, scapular spine pseudo-arthrosis was diagnosed. Scapular spine fracture could not be diagnosed for whole this time, and he was treated for soft tissue injury and operated for rotator cuff tear.

Some classification systems consider scapular spine fractures as an extension of acromion fractures [10]. Majority of these fractures can be treated with Kirschner wire and tension band wiring [10]. Scapular spine fractures that enter the spino-glenoid notch are different from isolated acromial fractures [3]. Therefore, they should be treated differently. For more proximal and medially displaced fractures involving the spine, plate fixation is more appropriate [3, 11].

For painful pseudo-arthrosis of the spine of the scapula, which is seen very rarely, operative treatment is indicated [12]. The few reported cases of pseudo-arthrosis of the spine of the scapula were treated by resection of the fibrous tissue, bone grafting of the fracture site and rigid fixation [1, 11]. In some cases, compression of the pseudo-arthrosis was established with one cancellous lag screw, without exposure, debriding or grafting. The screw was placed eccentrically to the upper part of the pseudo-arthrosis site, and this position resulted in a superior tilt of the acromion which made the impingement syndrome disappear [1].

Acute displaced symptomatic fractures distal to the acromial angle can be successfully treated with Kirschner wire and tension band wiring. For more proximal and medially displaced fractures involving the spine, plate fixation is more appropriate [10, 11].

We used plate fixation and grafting, after debridement of the pseudo-arthrosis site of the spine of the scapula. After a long period of mis-diagnosis, his pseudo-arthrosis could be managed. Symptoms of impingement regressed, and he had a pain-free arc of motion.

## Conclusions

If we remember the possibility of fracture or pseudo-arthrosis of the spine of the scapula and look for it on the roentgenograms, we do not miss the diagnosis. It is a rare fracture type, but we should not forget that it can be a reason of painful shoulder function for years. In order to prevent delayed or missed diagnosis of the spine of the scapula, just a simple roentgenogram and focusing on the scapula is enough.
